# Dietary diversity practice and associated factors among pregnant women in North East Ethiopia

**DOI:** 10.1186/s13104-019-4159-6

**Published:** 2019-03-07

**Authors:** Seid Aliwo, Melkitu Fentie, Tadesse Awoke, Zemichael Gizaw

**Affiliations:** 1 Oromia Special Zone Health Department, Amhara Region Health Bureau, Kemise, Ethiopia; 2 Department of Human Nutrition, Institute of Public Health, College of Medicine and Health Sciences, University of Gondar, Gondar, Ethiopia; 3 Department of Epidemiology and Biostatistics, Institute of Public Health, College of Medicine and Health Sciences, University of Gondar, Gondar, Ethiopia; 4 Department of Environmental and Occupational Health and Safety, Institute of Public Health, College of Medicine and Health Sciences, University of Gondar, Gondar, Ethiopia

**Keywords:** Dietary diversity, Pregnant women, Jille Tumuga, North East Ethiopia

## Abstract

**Objective:**

Diversified food during pregnancy is the very important since it is known to affect pregnancy and birth outcomes. The aim of this study was to assess dietary diversity practice and associated factors among rural pregnant women in North East Ethiopia.

**Result:**

A total of 647 pregnant women were participated with a response rate of 97.4%. The adequate dietary diversity practice of pregnant women was found to be 31.4% [95% confidence interval (CI) 27.8–35.2]. Cereals were the most commonly consumed food groups. Dietary diversity practice of pregnant women was associated with maternal education [Adjusted Odds Ratio (AOR) = 2.36, 95% CI 1.29, 4.32], wealth index (AOR = 1.85, 95% CI 1.21, 2.82), nutrition information (AOR = 2.51, 95% CI 1.05, 6.02) and Productive safety net program beneficiary (PSNP) (AOR = 1.7, 95% CI 1.16, 2.50). The dietary diversity practice of pregnant women was found to be low in the study area. Maternal education, wealth status, having nutrition information and PSNP beneficiary were the determinant factors.

## Introduction

Pregnancy is a critical period in the lifecycle during which additional nutrients are required to meet the metabolic and physiological demands as well as the increased requirements of the growing fetus [1]. So diversified dietary intake has to meet the needs of the mother as well as the products of conception [2]. Dietary diversity is the consumption of a variety of food groups over a reference period which has been accepted as an aspect of dietary quality and can indicate nutritional adequacy [3].

Diversified food during pregnancy is the most important since it is known to affect pregnancy and birth outcomes [4, 5]. The effect of inadequate intake of nutrients during pregnancy leads to irreversible damage to the fetus that can compromise the future work capacity and survival [6–8].

Diets of pregnant women in low and middle-income countries (LMICs) are monotonous, low quality and predominantly plant-based with little consumption of micronutrient-dense animal-source foods, fruits, and vegetables [9, 10]. The government of Ethiopia has launched National Nutrition Program and prioritized interventions like Promote maternal nutrition including adequate intake of diversified foods to improve the nutritional status of women. Even though the implementation of the above strategy, thinness and different micronutrient deficiencies are common problems during pregnancy [11, 12]. There is limited data regarding the dietary diversity practice and factors associated during pregnancy in Ethiopia in general and specifically in rural areas. Therefore, the purpose of this study was to determine the dietary diversity practice and associated factors among rural pregnant women in Jille Tumuga district, Oromia Special Zone, Amhara region, Northeast Ethiopia.

## Main text

### Method

A community based cross-sectional study was conducted at Jille Tumuga district from March to April 2017. Jille Tumuga is a rural district found 265 kms far from Addis Ababa, the capital of Ethiopia, 616 kms away from Bahirdar (the capital city of the region).. The district is divided into 18 rural kebeles (smallest administrative unit in Ethiopia) and there are four governmental health centers and 21 health posts. It has a predominantly kola (Tropical Zone) agro-ecology and the altitude of the district ranges from 1000 to 2000 m above the sea level. Cereals, such as maize, sorghum, wheat, and barley are the main staple crops cultivated in the district. The main livestock reared are cattle and goats.

All pregnant women who lived in Jille Tumuga district for at least 6 months were eligible for the study. Sample size was estimated using the single population proportion formula [13] by considering adequate dietary diversity as 50% (since there was no similar study in the study area), 1.5 design effect and 10% non response rate the required sample size was 634. Since cluster sampling technique was used, all pregnant mothers in the selected cluster were included and the final sample size was 664. Clusters were Kebeles and from total kebeles, 7 were selected by simple random sampling.

An interviewer administered structured and pre-tested questionnaire was used to collect socio-demographic, maternal and health related factors, feeding & dietary diversity practice, wealth index and food security status of pregnant women. It was first prepared in English and then translated to oromifa and translated back to English to maintain its consistency. To ensure the quality of data, pre-test was done and modifications were made accordingly. Six health extension workers and two diploma nurses were involved in data collection supervision respectively.

Tools for measuring dietary diversity was adopted from FAO guidelines for measuring minimum dietary diversity women, 2016. It was assessed by asking respondents to list all food items they consumed in the last 24 h preceding the survey day. A total of ten food groups were used and respondents who consumed < 5 food groups were classified as having inadequate dietary diversity whereas those consumed ≥ 5 food groups were classified as having adequate dietary diversity practice [14].

Household’s wealth index measuring tool was adopted from EDHS 2011 [15]. It was analyzed using Principal Component Analysis (PCA) by considering the household assets, such as livestock, type of house, durable assets, productive assets and agricultural land ownership. First, variables were coded between 0 and 1. Then variables entered and analyzed using PCA, and those variables having a communality value of greater than 0.5 were used to produce factor scores. Finally, the factor scores were summed and ranked into tertiles as poor, medium and rich.

Food insecurity was measured using FANTA household food insecurity access scale (HFIAS) tool. It is consisted of nine occurrence questions that represent a generally increasing level of severity of food insecurity (access), and nine “frequency-of-occurrence” questions. The frequency-of-occurrence question is skipped if the respondent reports that the condition described in the corresponding occurrence question was not experienced in the previous 4 weeks (30 days). Finally individuals were considered as food secure if they respond “no” to all of items or just experience worry but rarely; mildly food insecure if household worries about not having enough food sometimes or often/or unable to eat preferred foods; moderately food insecure if household scarifies quality more frequently, by eating a monotonous diet or undesirable foods sometimes or often and severely food insecure if household experience forced cutting back on meal size or number of meals often, and/or experiences any of the three most severe conditions [16].

The data was cheeked, coded and entered using EPI data 3.1 software and exported to SPSS version 20 statistical packages for further analysis. Data cleaning was performed. Frequencies and graphs were used to explore the data. Binary logistic regression was used to identify the confounders. Variables having p-value < 0.2 in the binary logistic regression was fitted into the multiple logistic regression models. Adjusted odds ratio (AOR) with 95% confidence interval (C.I) was computed to assess the presence and strength of association. Variables having p-value less than 0.05 in multiple logistic regressions were considered as significantly associated with the dependent variables. Hosmer–lemshow goodness of fit test was used for model adequacy checking and it was 0.52.

### Results

#### Socio-demographic characteristics

A total of 647 pregnant women were participated in this study with a response rate of 97.4%. The mean age of the participants was 26.21 (± 4.61) years. Most of the participants were married 643 (99.4%). Majority (68.3%) of the respondents were not educated. More than one third (33.2%) of the respondents were poor (Table 1).

**Table 1:** Socio demographic characteristics of pregnant women (n = 647) in Jille Tumuga district Northeast Ethiopia, 2017

Variable	Frequency (n)	Percent (%)
Age		
17–19	34	5.3
20–24	180	27.8
25–35	407	62.9
≥ 35	26	4
Marital status		
Married	643	99.4
Widowed	4	0.6
Religion		
Muslim	643	99.4
Orthodox	4	0.6
Ethnicity		
Oromo	576	89.0
Amhara	63	9.7
Argoba	8	1.2
Educational status of pregnant women		
No education	442	68.3
Read and write	60	9.3
Primary and above	145	22.4
Educational status of husband		
No education	381	58.9
Read and write	94	14.5
Primary and above	172	26.6
Occupation of pregnant women		
Housewife	638	98.6
Merchant	6	0.9
Government employee	3	0.5
Occupation of husband		
Farmer	614	94.9
Merchant	11	1.7
Government employee	6	0.9
Daily laborer	16	2.5
Family size		
1–2	71	11.0
3–4	365	56.4
> 5	211	32.6
Productive safety net program beneficiary		
Yes	275	42.5
No	372	57.5
Food security status		
Food secure	425	65.7
Mildly food insecure	18	2.8
Moderately food insecure	193	29.8
Severely food insecure	11	1.7
Wealth index		
Poor	215	33.2
Medium	216	33.4
Rich	216	33.4

#### Meal frequency and feeding practice

Less than half, 284 (43.9%) of respondents had meal frequency of four and above per day. Two hundred eighty six (44.7%) of respondents had the habits of taking snacks. One fifth of the respondents had habit of skipping meal and 158 (24.4%) of respondents had avoided some kind of food during pregnancy.

#### Dietary diversity practice of pregnant women

**Fig. 1:**
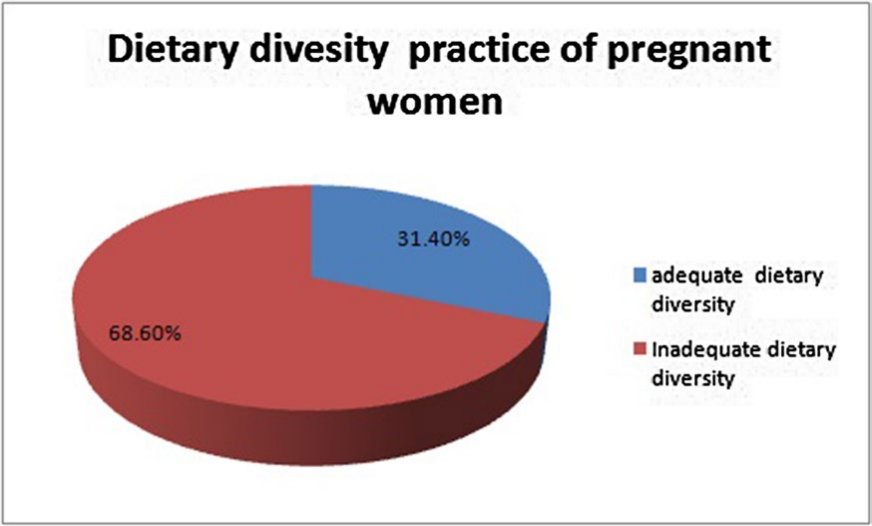
Dietary diversity practice among rural pregnant women during the preceding 24 h (n = 647) in Jille Tumuga district Northeast Ethiopia, 2017

The overall prevalence of adequate dietary diversity practice among pregnant women were found to be 31.4% with (95% CI 27.8–35.2%) (Fig. 1).

#### Factors associated with dietary diversity practice of pregnant

In the adjusted analysis mother’s educational status, wealth index, PSNP beneficiary and nutrition information were significantly associated with dietary diversity practices of pregnant women.

The odds of dietary diversity practice was 2.36 times (AOR = 2.36, 95% CI 1.29, 4.32) higher among pregnant women who can read and write than those who had no formal education. While the odds of dietary diversity practice was 94% (AOR = 1.94, 95% CI 1.24, 3.01) higher among pregnant women who had primary education and above than those having no formal education. With regard to wealth index, the odds of dietary diversity practice was 85% (AOR = 1.85, CI 1.21, 2.82) higher in rich pregnant women than the poor pregnant women. On the other hand, the odds of dietary diversity practice was 2.58 (AOR = 2.58, CI 1.05, 6.02) times higher among pregnant women who had nutrition information than their counterparts. While the odds of dietary diversity practice was 71% higher among pregnant women who are not beneficiary of productive safety net program than their counterparts (Table 2).

**Table 2:** Factors associated with dietary diversity practice of pregnant women in Jille Tumuga district, Northeast Ethiopia, 2017

Variable	Dietary diversity		COR with 95% CI	AOR with 95% CI
	Adequate n=203 (31.4%)	Inadequate		
Mothers educational status				
No education	119	323	1	1
Read and write	27	33	2.22 (1.28, 3.85)	2.33 (1.28, 4.26)*
Primary and above	57	88	1.75 (1.18, 2.60)	1.93 (1.24, 2.99)*
Husband education				
No education	126	255	1	1
Read and write	29	65	0.90 (0.55, 1.46)	0.78 (0.46, 1.34)
Primary and above	48	124	0.78 (0.52, 1.16)	0.70 (0.46, 1.09)
Wealth index				
Poor	61	154	1	1
Medium	49	167	0.74 (0.47, 1.14)	0.73 (0.46, 1.15)
Rich	93	123	1.90 (1.27, 2.89)	1.86 (1.22, 2.82)*
No of pregnancy				
< 3	113	250	1.46 (0.855, 2.505)	1.09 (0.57, 2.07)
4–5	69	126	1.77 (1.002, 3.137)	1.49 (0.80, 2.80)
> 6	21	68	1	1
ANC follow up				
Yes	8	42	1	1
No	195	402	2.54 (1.17, 5.53)	1.66 (0.61, 4.46)
Nutrition information				
No	10	55	1	1
Yes	193	389	2.73 (1.36, 5.47)	2.55 (1.06, 6.10)*
Nutritional status				
Undernourished	50	140	1	1
Normal	153	304	1.40 (0.96, 2.05)	1.42 (0.94, 2.12)
Age of pregnant women				
17–19	8	26	1.3 (0.37, 4.54)	1.51 (0.47, 4.88)
20–24	56	124	1.9 (0.68, 5.30)	1.88 (0.63, 5.59)
25–34	134	273	2.06 (0.76, 5.60)	
> 35	5	21	1	1
PSNP beneficiary				
Yes	64	211	1	1
No	139	233	1.96 (1.38, 2.79)	1.72 (1.17, 2.52)*

*indicates variables which were statistically significant in multi-variable analysis

### Discussion

Recognizing the dietary diversity practice of pregnant women is vital since it affects the health and long term productivity of the mother and the fetus. This study aimed at assessing the magnitude of adequate dietary diversity practice and its associated factors among pregnant women in Jille Tumuga district. The adequate dietary diversity practice of rural pregnant women found to be 31.4%. This finding is lower than the study conducted in Bangladesh 37% [17]; Togo 45% [18] and Northern Ghana 46.1 [19]. This difference may be due to socio demographic factors. In this study most of the participants had lower educational level compared with aforementioned studies, which could affect mother’s knowledge and practice of dietary diversity. Moreover, the study subjects of the current study had larger family size in which food sharing habit of large sized families is high and so that pregnant women may not get diversified foods as required. Most of the study participants of this study were also house wives whom could not generate money by themselves so it might affect their food purchasing power and further affect their dietary diversity practice.

This study showed that the odds of adequate dietary diversity practice was two and more times higher in pregnant women who can read and write than those having no formal education. While the odds of dietary diversity practice was 94% higher among pregnant women who had primary education and above than those having no formal education. This showed that when educational status of a pregnant women increased, their dietary diversity practice showed a significant advancement This finding is also supported by studies conducted in Nigeria, Rural Bangladesh, Kenya and Ghana [17, 20–23]. This might be due to the contribution of education in giving information about the importance of diversified diet consumption. On the other hand educated women can have better employment opportunity and income which can further improve their household food security status and consumption of diversified food.

According to this study dietary diversity was associated with wealth status of the household. There is increased odds of dietary diversity practice by 85% in rich pregnant women than the poor. This finding is in line with study conducted in Bangladesh, Ghana and Kenya [20, 23, 24]. This could be due to the fact that rich households will have access to a variety of food and the dietary practice of pregnant women in this household will be improved.

In addition, those who are not beneficiary of productive safety net program were 71% more likely to practice adequate dietary diversity than their counter parts. This might be due to PSNP is aimed for chronically food insecure households and these households food purchasing power and access to a variety of food is limited and cereal based monotonous diet is common.

This study revealed that having information about nutrition is significantly associated with the dietary diversity practice of pregnant women. The odds of dietary practice were 51% increased in those who got nutrition information than their counter parts. This is also supported by study conducted in East Wollega Zone, Ethiopia [25] and Gondar [26] in which nutrition information was important for good nutritional practices. This may be due to the fact that those who get information about nutrition will have better knowledge and understanding to practice diversified diet than those who do not.

Over all dietary diversity practice was low in the study area. Educational status of mothers, wealth status and nutrition information and PSNP beneficence were factors associated with adequate dietary diversity practice of pregnant mothers.

Multi-sectoral collaboration is needed to enhance the dietary diversity of pregnant women by promoting women’s education, strengthening sustainable income generating activities and saving strategies to improve the wealth status of pregnant women. In addition, it is better to advocate nutrition education regarding dietary diversity during pregnancy.

## Limitation

We didn’t consider the amount of food consumed that may not accurately reflect their past feeding experience. In addition, there might be social desirability bias during answering wealth index questions.

## Abbreviations

ANC: ante natal care
AOR: adjusted odd ratio
CI: confidence interval
COR: crude odd ratio
DDS: Dietary Diversity Score
EDHS: Ethiopia Demographic and Health Survey
EPI DATA: Epidemiological Data
FANTA: Food and Nutrition Technical Assistance
FAO: Food and Agriculture Organization
HFIAS: Household Food Insecurity Access Scale
LMICs: Low and Middle Income Countries
MDD-W: Minimum Dietary Diversity for Women of reproductive age
OR: odd ratio
PCA: Principal Component Analysis
PSNP: productive safety net program
SD: standard deviation
SGA: small for gestational age
SNNPR: South Nation Nationality Peoples Region
SPSS: Statistical Package for Social Science
UNICEF: United Nation International Children Emergency Fund
WHO: World Health Organization
